# Critical Assessment of Analytical Techniques in the Search for Biomarkers on Mars: A Mummified Microbial Mat from Antarctica as a Best-Case Scenario

**DOI:** 10.1089/ast.2016.1467

**Published:** 2017-10-01

**Authors:** Yolanda Blanco, Ignacio Gallardo-Carreño, Marta Ruiz-Bermejo, Fernando Puente-Sánchez, Erika Cavalcante-Silva, Antonio Quesada, Olga Prieto-Ballesteros, Víctor Parro

**Affiliations:** ^1^ Department of Molecular Evolution, Centro de Astrobiología (INTA-CSIC), Madrid, Spain.; ^2^ Department of Biology, Universidad Autónoma de Madrid, Madrid, Spain.

## Abstract

The search for biomarkers of present or past life is one of the major challenges for *in situ* planetary exploration. Multiple constraints limit the performance and sensitivity of remote *in situ* instrumentation. In addition, the structure, chemical, and mineralogical composition of the sample may complicate the analysis and interpretation of the results. The aim of this work is to highlight the main constraints, performance, and complementarity of several techniques that have already been implemented or are planned to be implemented on Mars for detection of organic and molecular biomarkers on a best-case sample scenario. We analyzed a 1000-year-old desiccated and *mummified* microbial mat from Antarctica by Raman and IR (infrared) spectroscopies (near- and mid-IR), thermogravimetry (TG), differential thermal analysis, mass spectrometry (MS), and immunological detection with a life detector chip. In spite of the high organic content (*ca*. 20% wt/wt) of the sample, the Raman spectra only showed the characteristic spectral peaks of the remaining beta-carotene biomarker and faint peaks of phyllosilicates over a strong fluorescence background. IR spectra complemented the mineralogical information from Raman spectra and showed the main molecular vibrations of the humic acid functional groups. The TG-MS system showed the release of several volatile compounds attributed to biopolymers. An antibody microarray for detecting cyanobacteria (CYANOCHIP) detected biomarkers from Chroococcales, Nostocales, and Oscillatoriales orders. The results highlight limitations of each technique and suggest the necessity of complementary approaches in the search for biomarkers because some analytical techniques might be impaired by sample composition, presentation, or processing. Key Words: Planetary exploration—Life detection—Microbial mat—Life detector chip—Thermogravimetry—Raman spectroscopy—NIR—DRIFTS. Astrobiology 17, 984–996.

## 1. Introduction

One of the outstanding scientific questions of our time is whether life has occurred on Mars. To date, most of the instruments devoted to organic detection in planetary missions only detected organic volatiles, such as the gas chromatograph–mass spectrometer (GC-MS) in the Viking missions (Biemann *et al.*, 1976), the Cassini/Huygens GC-MS (Israël *et al.*, 2005), the Phoenix Lander thermal evolved gas analyzer (Hoffman *et al.*, 2008), and the recent Sample Analysis at Mars (SAM) instrument suite onboard the Curiosity rover (Mahaffy, 2008; Summons *et al.*, 2011; Leshin *et al.*, 2013).

The European Space Agency (ESA) ExoMars mission (Vago *et al.*, 2015) plans to drill 2 m into the martian subsurface and analyze samples with a Raman spectrometer and the Mars Organic Molecule Analyzer (MOMA) (Siljeström *et al.*, 2014) instruments. The Raman spectrometer will characterize the mineralogical composition and potentially detect some organics in a nondestructive way (Rull *et al.*, 2011; Böttger *et al.*, 2012). The MOMA instrument consists of three complementary operational modes: pyrolysis-GC-MS, derivatization-GC-MS, and LD-MS (laser desorption– mass spectrometry). Volatile organic compounds can be detected directly by GC-MS after pyrolysis, the derivatization process makes accessible other less volatile ones for GC-MS, and the LD-MS mode will detect larger and heat-resistant materials (Arevalo *et al.*, 2015).

Assigning biological information to any organic matter found might be a cumbersome task because most of it is nonvolatile and recalcitrant and it is partially destroyed or altered under the experimental conditions of these instruments (Benner *et al.*, 2000). The effect is even higher after the strong destructive effects at high temperature of perchlorates and other oxidants found on the surface of Mars (Hecht *et al.*, 2009; Kounaves *et al.*, 2010; Glavin *et al.*, 2013; Leshin *et al.*, 2013). Therefore, characterizing martian organics requires the development of new techniques that are compatible with martian soil chemistry.

Methods based on wet chemical extraction and further analyses with biosensors are being considered for planetary exploration (Parro *et al.*, 2005; Sims *et al.*, 2005). The Signs Of LIfe Detector (SOLID) instrument concept (Parro *et al.*, 2005, 2008, 2011b) is an antibody microarray-based biosensor capable of detecting molecular biomarkers extracted from soil, rock, or ice. SOLID can extract the organics from soil or rock powder into a liquid solution by a process that involves ultrasonication at temperatures below 100°C. The liquid extract is then analyzed by the core part of the instrument, the Life Detector Chip (LDChip), by fluorescent sandwich immunoassay (Rivas *et al.*, 2008; Parro *et al.*, 2011b, c; Fernández-Remolar *et al.*, 2013). For an appropriate performance, this and all the above-mentioned techniques require that their target biomarkers are accessible to the analytical device and have a certain degree of preservation.

Cyanobacterial-driven microbial mats growing in the polar regions under low temperature, water scarcity, and high UV radiation are considered modern analogues of early microbial ecosystems as well as terrestrial analogues for hypothetical past martian communities (Laybourn-Parry and Pearce, 2007). After a long dry period, the entire mat experiences a *mummification* process that can preserve it for an extended period of time, as described in other environments exposed to desiccation (Golubic, 2000). The apparent absence of biological activity and the age of these mats make them a good proxy for studying hypothetical ancient martian analog structures that might have been preserved after similar desiccation processes. Therefore, we subjected an old mat sample to a comparative analysis with several techniques already used or proposed for organic detection in planetary missions.

The aim was to compare the information obtained with the different techniques from a best-case scenario sample, make a critical assessment, and establish a procedure or guidelines to use a suite of complementary *in situ* techniques for organic and life detection in planetary exploration. The instrumentation was selected according to an increasing order of information they could provide: mineralogy and biological pigments (Raman), organic and chemical bonding (IR), molecular structures (TG-MS), and biochemical/biological compounds (CYANOCHIP). Fluorescent microscopy, biochemistry, and DNA analyses were carried out as ground truth techniques.

## 2. Experimental Procedures

### 2.1. Site description

The McMurdo Ice Shelf is the largest single area of nonmarine biomass in southern Victoria Land (Antarctica) (Kellogg and Kellogg, 1987; Vincent, 1988). The Ice Shelf ablation is a 1500–2000 km^2^ region of marine-derived ice that floats on the sea and is covered by numerous meltwater ponds, streams, and small lakes (Howard-Williams *et al.*, 1989), most of which are colonized by thick cyanobacterium-dominated mats (Vincent *et al.*, 1993).

### 2.2. Sample collection and processing

**FIG. 1.**
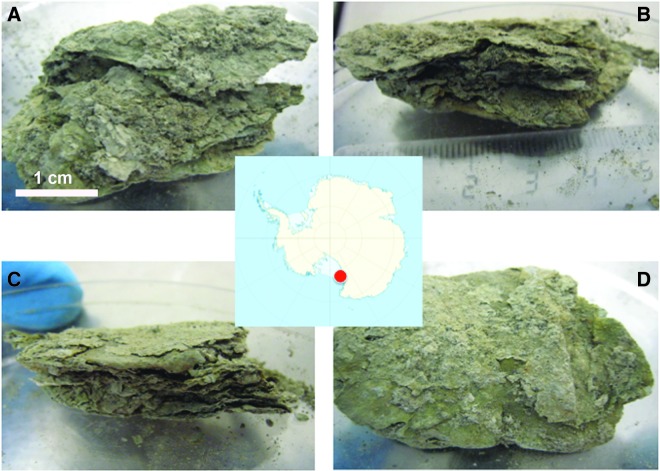
An old and *mummified* microbial mat from the McMurdo Ice Shelf (Antarctica). Pictures of the microbial mat showing the top part of Sample A **(A)**, its laminar structure **(B, C)**, and the bottom part **(D)**. In **(B)**, top part of the photograph corresponds to the top part of the sample. In **(C)**, top part of the photograph corresponds to the bottom part of the sample. The ruler in **(B)** indicates cm scale.

A dry, *mummified* microbial mat (Sample A) was collected in the McMurdo Ice Shelf ablation zone during the summer season of 1996. This mat corresponded to an apparently old material found at the top of the hills at around 30–40 m distance from the actual lake level. It was collected in sterile Whirl-Pak plastic bags, stored at room temperature in the dark, and left unopened until the development of the experiment in 2015 under sterile conditions. The sample was cut with a razor blade into three sections of 0.5 cm thickness, top (A1), middle (A2), and bottom (A3) parts (Fig. 1).

### 2.3. Radiocarbon dating

Up to 20.1 mg of a mixture of A1 and A2, 0.5-cm-thick transverse sections of Sample A (the oldest parts of the mat), was taken for ^14^C-AMS (Accelerator Mass Spectrometry) dating by Beta Analytic, Inc. (Miami).

### 2.4. Biochemical analysis: protein and carbohydrate content

Total protein and sugar content in Sample A was determined as follows: 0.5 g of a mixture of A1, A2, and A3 was suspended in 1 mL of distilled water and subjected to 5 × 1-min ultrasonication cycles, with 1-min stops on ice between each cycle, by using a manual sonicator (Dr. Hielscher 50W DRH-UP50H sonicator; Hielscher Ultrasonics, Berlin, Germany). The sample was filtered through a 20-μm filter, and filtrate was directly assayed for protein as described by Bradford (1976) and carbohydrate quantification as described by Dubois *et al.* (1956) using bovine serum albumin (BSA) and glucose as standards, respectively.

### 2.5. Analysis by fluorescence microarray immunoassays

Sample A was analyzed with CYANOCHIP, an antibody microarray for detecting the most relevant cyanobacterial genera of both planktonic (*Anabaena*, *Aphanizomenon*, *Microcystis*, *Planktothrix*) and benthic (*Chamaesiphon*, *Leptolyngbya*, *Nostoc*, *Phormidium*, *Rivularia*, *Tolypothrix*) habitats (Blanco *et al.*, 2015). Aliquots of Sample A were analyzed by a sandwich microarray immunoassay, as described previously (Blanco *et al.*, 2015). Briefly, 0.1 g of each transversal section of the mat was ultrasonicated in 1 mL of TBSTRR buffer (0.4 M Tris HCl pH 8, 0.3 M NaCl, 0.1% Tween 20) as above and filtered through a 20-μm filter. From 10 to 20 μL of the filtrate was incubated with the CYANOCHIP set up onto a 3 × 8 chamber hybridization cassette (Arrayit.com) in a final volume of 50 μL of TBSTRR buffer for 1 h at room temperature. After a wash step with TBSTRR, immunoreactions were revealed by adding a mixture of 17 fluorescent anticyanobacterial antibodies (Alexa-647) at 0.7 μg/mL for 1 h at 4°C. After a final wash, the slides were dried and scanned for fluorescence at 635 nm in a GenePix 4100A scanner.

Blank assays were run in parallel using buffer only and revealed with the same fluorescent antibody mixture. BSA, printing buffer, and the preimmune antiserum samples were used as control spots in the microarray. An additional control was performed to verify the authenticity of antigen–antibody reactions; 0.1 g of each sample section was heated at 300°C for 3 h in porcelain pots and analyzed as above. The heat should destroy the structure of the biomolecules and cause the fluorescent signal to disappear.

Fluorescent images were analyzed and quantified by Genepix Pro Software (Genomic Solutions). The final fluorescence intensity A of each antibody spot was calculated with the equation: A = (F635-B)_sample_−(F635-B)_blank_, where F635-B is the fluorescence intensity at 635 nm minus the local background around the spots. Up to 2.5 times the average fluorescence of all the spots on the microarray was used as a baseline cutoff to minimize false positives.

### 2.6. Thermal analysis

Thermogravimetry (TG), derivative TG, and differential thermal analysis measurements were performed with a simultaneous thermal analyzer model SDTQ-600/Thermo Star of TA Instruments. Nonisothermal experiments were carried out under dynamic conditions from room temperature to 1000°C at a heating rate of 10°C/min under argon and oxygen atmospheres. The average sample weight was ∼10 mg, and the argon and oxygen flow rate was 100 mL/min. A coupled TG-MS system with an electron impact quadrupole mass-selective detector (model Thermostar QMS200M3) could analyze the main species evolving during the dynamic thermal decomposition of fragmentation processes of sections A1, A2, and A3 from the dry mat.

### 2.7. Raman spectroscopy

No sample preparation was needed for Raman spectroscopy analysis. Raman spectra were taken directly from each section of the microbial mat, as expected for near-future missions to Mars (*e.g.*, ExoMars). Raman spectra were stimulated with a 532 nm solid-state laser, completely unpolarized, and with reduced power (200 mW) to minimize sample alterations. After focusing onto a monochromator (Horiba JobinYvon HRi550, 550 mm optical length), with a diffraction grating of 600 grooves/mm, the scattered light was detected with a charged couple device cooled to 203 K for thermal noise reduction. Optical fibers with diameters of 100 and 50 μm, respectively, were used to connect both the spectrometer and the laser to the optical probe head. Pixel resolution of the equipment was 2.16 cm^−1^/pixel (binning factor = 1). Spectral resolution was better than 10 cm^−1^ for simulating the conditions of space instruments.

### 2.8. Diffuse reflectance infrared Fourier transform spectroscopy

Diffuse reflectance infrared Fourier transform spectroscopy (DRIFTS) was applied on powdered samples of the dry mat. We used a Thermo Nicolet Nexus spectrometer working with a Diffuse Reflection Praying Mantis™. A DTGS (deuterated triglycine sulfate)-KBr detector and XT-KBr beam splitter were selected to cover the wavelength range from 1 to 10 μm (10,000 to 100 cm^−1^). Measurements were performed with 128 scans, spectral resolution better than 4 cm^−1^, gain 1, aperture 60, and mirror velocity of 0.1581.

### 2.9. X-ray diffraction

X-ray diffraction (XRD) of the powdered samples was performed by a Seifert 3003 TT with Cu Kα anode (*λ* = 1.542 Å). The X-ray generator was set to an acceleration voltage of 40 kV and a filament emission of 40 mA. Range of measurement is 5 to 60°, stepscan 0.1° every 2 s. In addition, oriented aggregate samples were used to determine the fine grain mineralogy.

## 3. Results

### 3.1. The mineralogical and organic context with Raman and IR spectroscopies

The desiccated microbial mat under study (Sample A) had a light brown and gray color and no photosynthetic pigments were apparent (Fig. 1). The laminar structure was still visible, with a similar aspect to cardboard. Conventional radiocarbon analysis estimated an average age of 1070 years. The mineral content accounted for 80% of the mass, with the total organic carbon around 20% (wt/wt).

**FIG. 2.**
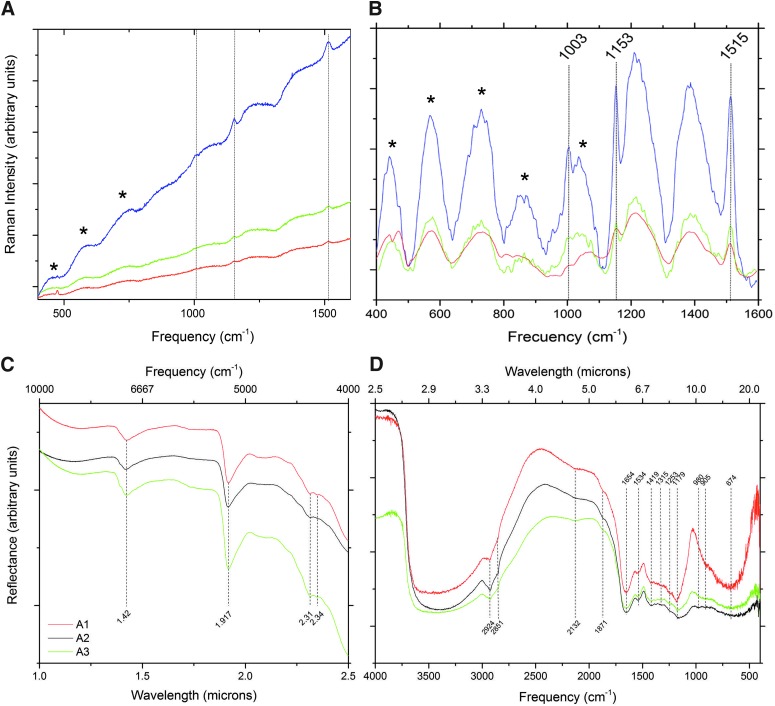
Raman and IR spectroscopy detected molecular biomarkers. Raman spectra **(A)** from the three sections of Sample A (A1, red; A2, green; and A3, blue) showing the three characteristic peaks of beta-carotene pigment (vertical lines). **(B)** Fluorescence baseline-corrected Raman spectra. Asterisks in **A** and **B** indicate the most relevant peaks assigned to Si-O-Si mode phyllosilicates, mostly montmorillonite, and corresponded to 430, 568, 720, 860, and 1035 cm^−1^. DRIFTS spectra in NIR **(C)** and MIR ranges **(D)** of the same sections of Sample A showing the most relevant spectral peaks. See the text for details and band assignations. DRIFTS, diffuse reflectance infrared Fourier transform spectroscopy.

To investigate any specific characteristic along the vertical profile, Sample A was fractionated into three 0.5-cm-thick sections from top to bottom (A1, A2, and A3). Fine grain-sized minerals, visually like clays, dominated all the transversal sections. Raman spectroscopy showed weak Raman scattering in a high fluorescence background (Fig. 2). Faint spectral peaks at 430, 568, 720, 860, and 1035 cm^−1^, characteristic of the Si-O-Si modes of phyllosilicates, mostly montmorillonite, were observed only after fluorescence background subtraction. The presence of other phyllosilicates, such as saponite, was confirmed by NIR spectral peaks at 1.42, 1.917, 2.31, and 2.34 μm (Fig. 2). Some spectral peaks indicated mineral hydration, such as those at 1.91 and 2.31 μm, characteristic of Mg-Fe smectites. Other NIR signatures at 2.71 and 2.69 μm (3679 and 3717 cm^−1^), and in MIR (mid infrared) at 9.7 and 15.1 μm (660 and 1030 cm^−1^), also supported the presence of that clay. Oriented aggregate protocols in XRD analysis confirmed the Raman and IR mineralogy results, showing the presence of phyllosilicates, such as saponite, montmorillonite, and palygorskite. Additionally, X-ray diffractographs indicated the presence of minerals derived from volcanic rocks such as Na-Ca-Fe pyroxenes (augite) and Ca-plagioclases (anorthite) (Data not shown).

Organic matter was detected with the Raman spectrometer after lowering the laser power to minimize the damage of the molecules and subtracting the background fluorescence. Particularly, a three-peak pattern (at 1515, 1153, and 1003 cm^−1^) identified the biological pigment β-carotene. Interestingly, the spectral peaks were more prominent in the lower section of the sample, presumably due to better preservation of pigment molecules (Fig. 2A, B).

DRIFTS identified several organic functional groups attributed to humic substances (Naumann *et al.* 1988, 1991) (Fig. 2C, D). No significant differences were found between the vertical sections (A1, A2, and A3) of the sample. The assignment of the main peaks to several vibrational functional groups of organics (frequency cm^−1^ [assignment]) was 3401 region [O-H stretch, with potential contribution of N-H stretch], 2960 [C-H asymmetric stretch (CH_3_)], 2921 [C-H asymmetric stretch (CH_2_)], 2854 [C-H symmetric stretch (CH_2_)], 2119 [unknown], 1650 [amide I: C = O, C-N, N-H], 1540 [amide II: N-H, C-N], 1423 [C-H bending], 1315 [ester C-O], 1168 [C-OH asymmetric stretch], 997 [Si-O stretching], 863 [possibly the aromatic C-H out of plane bend], and 667 [O-H bending]. The presence of amide I and II was potentially due to peptides from proteins. Peaks at 2960 and around 1000 cm^−1^ probably indicated the presence of polysaccharides.

### 3.2. Detection of volatile compounds by thermal analysis

Thermal volatilization and pyrolysis are the most used systems for sample preparation for detecting organic molecules in planetary exploration. Sample A was also subjected to thermogravimetry analyses to extract as much information as possible of its organic content. The thermogravimetric patterns obtained for the three sections of the mat indicated a similar behavior, although with little differences (data no shown). The thermal degradation of the different sections was divided into four stages, with a total mass loss around 18, 16, and 24 wt % for A1, A2, and A3, respectively, when they were heated under Ar.

The effect of oxygen on thermal degradation of the three sections was also analyzed. A1 and A3 showed a thermal degradation that was divided into four stages with a total mass loss of 21 wt % in both cases. The degradation of A2 also indicated a total mass loss of 21 wt %, but in three steps. This total mass loss indicated that around 80 wt % of A1 and A2 was inorganic substances and that the organic compounds after Ar heating led to the formation of ca. 3 wt % and 5 wt % of graphitic material, respectively. In the case of A3, the lower total mass loss in the presence of oxygen could be due to the formation of nonvolatile oxides.

**FIG. 3.**
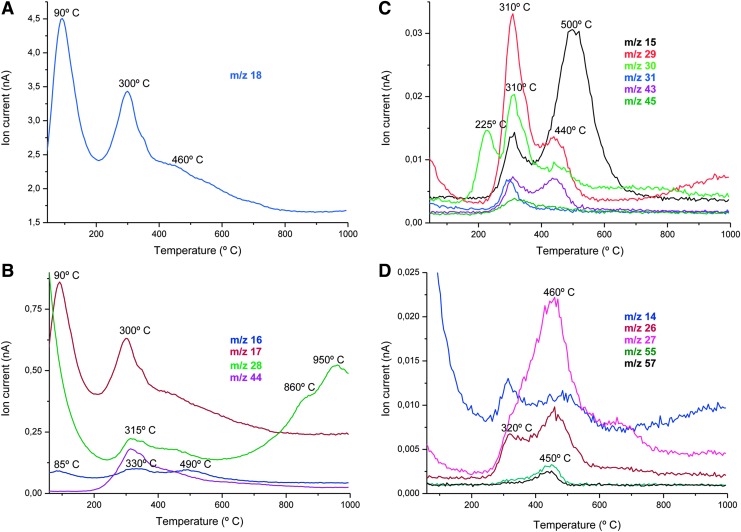
Thermal volatilization and mass spectrometry analysis. Evolution of the TG-MS curves for Sample A (A3) from 25°C to 1000°C. The main processes observed in thermal decomposition of samples are dehydroxylation, dehydration, and decarboxylation. **(A)** m/z 18, water; **(B)** m/z 16, O^+^ and CH_4_^+^; m/z 17, OH^+^; m/z 28, carbon monoxide; m/z 44, carbon dioxide; **(C)** m/z = 15, 43, 45 (CH_3_COO^+^, ion acetate); m/z = 29 (CHO^+^, methanal); m/z = 30 (HCHO^+^, ethanol and NO); m/z = 31 (CH_3_O^+^, methanol and/or hydroxyacetaldehyde); m/z = 43 (CH_3_CO^+^), m/z = 45 (COOH^+^); **(D)** Peaks related to hydrocarbons (m/z 14, 26, and 27 and also m/z 55 related to alkanes and m/z 57 alkenes). MS, mass spectrometry; TG, thermogravimetry.

MS was coupled to the TG system to study the volatile species after thermal decomposition and fragmentation processes in the three sections. All of them showed similar MS profiles for gases (Fig. 3 shows the TG-MS curves for A3). The most intense peak appeared at 90°C and 300°C with m/z = 18, which corresponded to H_2_O^+^ (Fig. 3A). The second major signal corresponded to m/z = 17, which was attributed to OH^+^ (Fig. 3B). The high amount of adsorbed water and likely crystallization of water of inorganic compounds in the samples resulted in a double water peak. In addition, these peaks showed a shoulder at higher temperatures that could be related to the dehydroxylation process. The other two main peaks, m/z = 28 (CO^+^) and m/z = 44 (CO_2_^+^) (Fig. 3B), with a maximum around 300°C indicated notable decarboxylation processes, and the maximum at 860°C and 950°C in the peak m/z = 28 could be related to decomposition of carbonates. Therefore, the heating of all Sample A sections led mainly to dehydration and decarboxylation processes.

Several volatile compounds detected by TG-MS (Fig. 3C) were related to the decomposition of polysaccharides: m/z = 15, 43, 45 (CH_3_COO^+^, ion acetate); m/z = 29 (CHO^+^, methanal); m/z = 30 (HCHO^+^, ethanol); m/z = 31 (CH_3_O^+^, methanol and/or hydroxyacetaldehyde); m/z = 43 (CH_3_CO^+^); m/z = 44 (CO_2_^+^); and m/z = 45 (COOH^+^). The TG-MS curve for m/z 30 showed three components, the first of which with a maximum at 225°C could be associated with NO from the decomposition of nitrates/nitrites of the inorganic substrate. Organic volatiles associated with hydrocarbons were also detected (Fig. 3D) and several peaks (m/z 14, 26, and 27 and also m/z 55) were related to alkanes and others (m/z 57) to alkenes. Finally, the masses m/z 12, 15, 17, 18, 28, and 44 could also be related to thermal decomposition of protein/peptides. In summary, TG-MS analysis showed complex patterns of organics that might come from the thermal decomposition of large polymers such as proteins and polysaccharide material present in the desiccated mat.

### 3.3. Detection of cyanobacterial markers with an LDChip

**FIG. 4.**
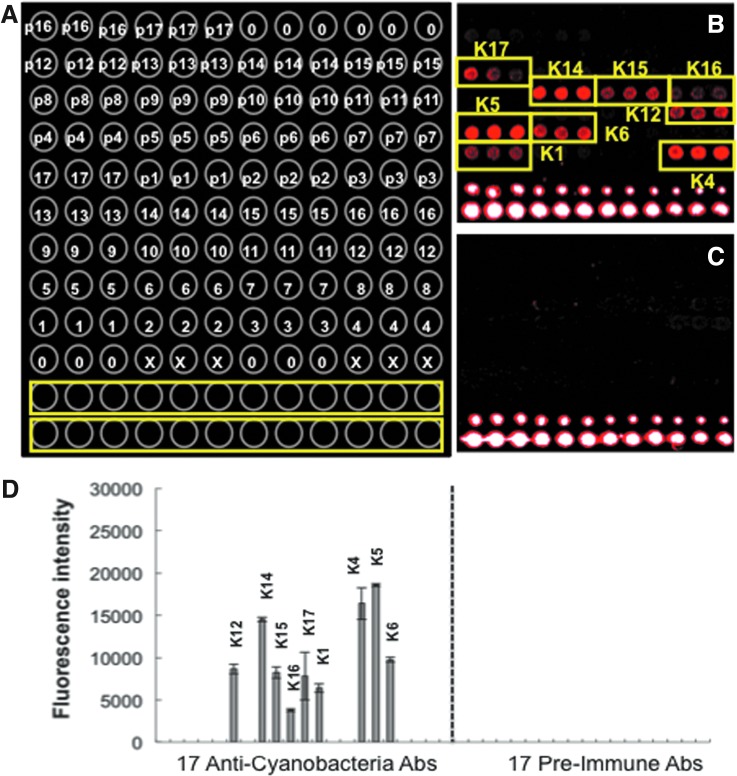
CYANOCHIP detected cyanobacterial markers in a *mummified* Antarctic microbial mat. (**A)** Schematic of a printing pattern layout (in triplicate) of the anticyanobacterial antibody collection on CYANOCHIP: (o) BSA, bovine serum albumin; (x) only printing buffer; (1–17) each of the antibodies as in Table 1 in the work by Blanco *et al.* 2015 (K1 to K17). From p1 to p17, the corresponding preimmune antibodies as controls. Yellow rectangles correspond to a fluorescent spot gradient as a reference. Image **(B)** and immunogram **(D)** showing the fluorescence intensity of the positive spots after analysis of 50 μL of extracts prepared from 0.1 g of Sample A (A3) with CYANOCHIP by fluorescence immunoassay. **(C)** The corresponding extract from Sample A (A3) heated at 300°C for 3 h.

Because Antarctic microbial mats are normally dominated by cyanobacteria, we searched for cyanobacterial markers in the three sections of Sample A by fluorescence sandwich microarray immunoassay with the CYANOCHIP (Experimental Section). No differences were observed between the three sections (Fig. 4). High fluorescence intensity was recorded with antibodies produced to benthic cyanobacterial species isolated from Antarctic mats such as *Anabaena* sp. and *Leptolyngbya* sp. (K14 and K15), and with antibodies to planktonic cyanobacteria such as *Microcystis* spp. (K4 and K5), *Aphanizomenon* spp. (K6 and K12), and *Planktothrix rubescens* (K17). Low, although positive, signals were obtained with antibodies to *Anabaena* PCC7120 (K1) and other benthic species isolated from an Antarctic mat as *Tolypothrix* sp. (K16). Altogether, the CYANOCHIP revealed the presence of multiple polymeric cyanobacterial markers, in spite of the age and advanced desiccated and mummified state of Sample A.

As the ground truth, biochemical and DNA analysis corroborated the presence of a complex chemistry of biological origin. The sample contained 0.86 and 7.8 mg/g of total proteins and carbohydrates, respectively. These data indicate that Sample A contained abundant complex biological matter—biopolymers and other cell remains—(which is in agreement with the results obtained when using the different techniques described above) that could be a target for the antibodies in the CYANOCHIP. Additionally, we extracted up to 1 μg of total DNA per gram of sample. Although sequencing the bacterial 16S RNA gene revealed the presence of a heterotrophic microbial community (to be published elsewhere), not a single sequence attributed to cyanobacteria was retrieved. The absence of amplifiable DNA suggests that the nucleic acids from these microbes were destroyed by UV radiation during desiccation and mummification processes.

## 4. Discussion

### 4.1. Desiccated Antarctic microbial mats as a best-case scenario for well-preserved potential martian microbiology

Although the temporal relevance of the *ca*. 1000-year-old microbial mat analyzed herein is very limited with regard to geological time scales, either for Earth or for Mars, its high degree of preservation would represent a best-case scenario for hypothetical martian biofilms that followed similar processes under similar environmental conditions: freezing temperatures, extremely low humidity, and high UV radiation.

Liquid water environments on Mars (*e.g.*, Saper and Mustard, 2013) and ancient martian seas (Rodriguez *et al.*, 2016) might have been the scenario for microbial biofilm development billions of years ago. Even more recent biological activity might have occurred in Mars near-surface ice during the multiple high obliquity periods that occurred over Mars' history, particularly during the last 10 My (Head *et al.*, 2003; Laskar *et al.*, 2004; Stoker *et al.*, 2010). Approximately every 125,000 years (Laskar *et al.*, 2004), water-rich northern lowlands receive high insolation from the Sun, which facilitates warmer summers and higher atmospheric humidity. During high obliquity, surface and near-subsurface temperatures experienced by ice deposits above 60° North are compatible with microbial growth and repair (Zent, 2008). With climate change, martian seas and lakes might have undergone desiccation processes similar to those in the Antarctic lakes and ponds, leaving mummified/lyophilized microbial mats that in turn might have been protected from radiation damage underneath regolith layers or frozen in ice for thousands or even millions of years.

The extremely low temperatures, minimal liquid water availability, and high UV radiation make some Antarctic environments the best terrestrial analogues for most of the current martian surface and near-subsurface (Tamppari *et al.*, 2012). On the McMurdo Ice Shelf, there are many lakes and ponds colonized by benthic algae and microbial mats that experience partial or complete water loss for long periods of time (Jungblut and Neilan, 2010). The extreme cold and progressive desiccation conditions drive the microbial mats to a state similar to a natural lyophilized status or a sort of mummified status. Under such conditions, many biopolymers and other biochemical compounds can be very stable for long periods of time (García-Pichel *et al.*, 2013). How long this *mummified* microbial mat could remain recognizable is unknown, although some old communities comprising laminated cyanobacterial mats have been found in polar regions that have been retained in permafrost for over a million years (Vishnivetskaya *et al.*, 2005).

The fact that no examples of cyanobacterial DNA were isolated could be due to limited DNA repair mechanisms at low temperatures in cyanobacteria (Vincent, 2007), which together with the high UV radiation and progressive desiccation might have damaged DNA for further polymerase chain reaction (PCR) amplification and sequencing. This is in agreement with the results obtained by Baqué *et al.* (2016) where the DNA extracted from a cyanobacterial mat exposed to UV radiation was not PCR amplifiable unless it was protected with minerals. Although the sample was collected 19 years before the analysis, it was stored in the dark and under sterile conditions, so any effect from external microbes, oxidation, or humidity can be discarded. The only effect that might be expected during this period is either a minimal increase of organic matter degradation or perhaps a negligible native heterotrophic microbial activity. Considering the storage conditions, we do not expect any significant alteration of the sample.

### 4.2. A suite of analytical techniques for increasing molecular complexity in planetary exploration

The performance of a suite of analytical techniques used or proposed for planetary exploration was critically assessed in the search for organics on a desiccated microbial mat as a best-case scenario. A summary that indicates the obtained information following increasing complexity is shown in Table 1. From mineralogy to biochemistry, we detected a broad range of organic matter, from simple chemical functional groups, low-molecular-mass compounds, to microbial pigment and unequivocal cyanobacterial markers in a sample with 80% mineral fraction and 20% organic matter.

**Table 1.** An Instrument Suite for Life Detection and Sample Characterization in Planetary Exploration

*Technique*	*Measurement*	*Relevance*	*Evidence of life?*
XRD and NIR	Mineralogy: Phyllosilicates, Pyroxene, plagioclases	Sedimentary context. Possible organic–mineral interactions, protection of the organic matter	No
Raman	High background. Phyllosilicates and pigments detected	Relevant sample that may contain organic matter and a biomarker: beta-carotene.	No
DRIFTS (MIR)	Organic chemistry, chemical bonds, and functional groups: C-H, C=C, C-N	Diverse organic matter associated with certain minerals. Chemical diversity and potential biochemistry	Possibly. Unequivocal signatures from proteins must be found
TG/MS	Water, acetic acid, alcohols, aromatic compounds, hydrocarbons?	Complex organic chemistry. Possibly biological polymers: proteins, polysaccharides	Possibly. Unequivocal signatures from biopolymers must be found
SOLID-LDChip	Cyanobacterial biomarkers with phylogenetic affiliation: *Nostocales*, *Oscillatoriales*	Confirm complex biology. Phylogenetic affiliation and ecosystem features: benthonic, planktonic	Yes. Biochemical and biological 3D structures found. Microbial markers

A summary of the different techniques used in this work and the obtained information following increasing complexity (from top to bottom).

3D = three-dimensional; DRIFTS = diffuse reflectance infrared Fourier transform spectroscopy; LDChip = Life Detector Chip; MS = mass spectrometry; SOLID = Signs Of LIfe Detector; TG = thermogravimetry.

The fact that two important scheduled missions to Mars, ESA's ExoMars (Rull *et al.*, 2011) and NASA's Mars 2020 (Beegle *et al.*, 2015; Wei *et al.*, 2015), include miniaturized Raman spectrometers to study the mineralogy and implement their capability to detect organic matter led us to pay special attention to the performance of this technique on the analysis of Sample A. Although the Raman spectrometer detected phyllosilicates, it exhibited some important limitations for obtaining more detailed information. The mineralogical context of the sample could be a worst-case scenario for Raman analysis because the targets mainly consisted of a low crystalline clay mineral with small grain sizes. Clays are very weak Raman scatterers, principally due to three causes: (1) the fine particle size (<2 μm); (2) the low degree of crystallinity, which depends on the formation processes; and (3) the presence of clays and bulk organic matter, which produce a high fluorescence background that obscures the organic spectral features.

Hooijschuur *et al.* (2016) showed how the strong background fluorescence could be reduced when making use of gated detection in time-resolved Raman spectroscopy and they detected pigments under mineral backgrounds of translucent calcite and transparent halite. Alternatively, changing the excitation laser wavelength sometimes can reduce fluorescence. The 532 nm wavelength was selected as the Raman setup for ExoMars; however, our results herein indicate that it could be cumbersome in samples with high clay content. Further analysis by Fourier transform or time-resolved Raman spectroscopy might be considered in an effort to work around the high fluorescence, but these sophisticated tools will not be available for near-future space exploration applications. Consequently, the expected performance of this technique on Mars must face these limitations, especially in clay-rich landing sites proposed (Bridges *et al.*, 2016) for future missions.

The versatility of Raman spectroscopy has permitted its use for detecting specific molecular spectral biosignatures from microbial pigments such as carotenoids or chlorophylls when the target is well differentiated from the mineral matrix (Edwards *et al.*, 2005; Böttger *et al.*, 2012). However, most of the work that has been reported was done with relatively concentrated targets, such as lichens on natural rocks, or biological pigments in solutions (Winters *et al.*, 2013; Maia *et al.*, 2014). In Sample A of this work, the Raman system was able to detect, only after lowering the laser power, β-carotene in the three vertical sample sections, which generated higher spectral peaks in the top section than in the lower ones. This gradient can be explained by the destructive effect of the UV radiation the sample has been subjected to for 1000 years with no repair by photosynthesis. In fact, this is the effect that Baqué *et al.* (2016) reported after exposing pure cyanobacterial mats to several UV radiation doses. The Raman system easily detected the pigments, but after strong UV radiation, the characteristic peaks disappeared and the Raman system ceased to detect them, even when most of the cellular content was still present.

Similarly, Dartnell *et al.* (2012) reported the destructive effect of ionizing radiation on Raman biosignatures for cellular carotenoids (deinoxanthin and β-carotene) of two model organisms, the cyanobacterium *Synechocystis* sp. PCC 6803 and the extremely radiation-resistant *Deinococcus radiodurans*. This effect of radiation is critical and may jeopardize the Raman system performance for detecting organics in a true Mars scenario, where organic content of the sample is expected to be much lower than in our sample and much more affected by UV and cosmic radiation.

For these kinds of altered samples, DRIFTS is the more appropriate setup with which to identify the organic matter chemistry. In fact, a rich and complex biochemistry can be inferred from the DRIFTS spectra and the stretch peaks that were assigned to several functional groups (Fig. 2). However, while this technique is highly valuable in the laboratory, the significant absorption of the martian atmosphere in the MIR range precludes its use for *in situ* measurements.

Although they have a relatively low signal-to-noise ratio, the thermal volatilization methods similar to those used for *in situ* planetary exploration provided relevant information about the biochemical nature of the sample. The most significant TG-MS curves obtained indicated that the main thermal decomposition processes were dehydration and decarboxylation (Fig. 3). However, other TG-MS curves with minor intensity were observed that were mostly related to the presence of polysaccharides and proteins that in fact were the main organic components of the sample as indicated by biochemical data. The intensity of TG-MS curves is related to the main volatiles released from the sample and is comparable with curves obtained with synthetic and pure organic samples generated during prebiotic chemistry experiments, such as tholins and HCN polymers (De la Fuente *et al.*, 2011; Nna-Mvondo *et al.* 2013; He *et al.* 2015).

From TG-MS results, a complex chemistry was inferred with products and functional groups associated with protein and polysaccharides. The low signal-to-noise ratio of all peaks associated with organic molecules is remarkable, considering that this sample is 20% organic species. Martian organics are, in general, likely to be in very low concentration; in fact, Freissinet *et al.* (2015) reported up to 300 ppb of chlorobenzene at Gale crater. This fact highlights the difficulty of extracting biological information from dirt samples by way of remote *in situ* instrumentation. The sample analyzed in the present study consisted mainly of polymeric material that could make the volatilization process highly inefficient for two reasons: (1) the inherent difficulty of detaching a simple compound from polymers and (2) the loss of biological information due to high fragmentation of released compounds. Regardless, these are conditions and constraints that any instrument will encounter during *in situ* planetary exploration. Our results emphasize the need for further improvement of the technique either by the incorporation of a new sample pretreatment protocol or preconcentration procedures, or by increasing the sensitivity of the instruments.

The use of bioaffinity-based sensors such as antibodies or antibody-like molecules for detection of biomarkers in astrobiology is compatible with martian soil chemistry and independent of the type and properties of the substrate (Parro *et al.*, 2011a). The CYANOCHIP showed strong positive signals (Fig. 4) for antibodies to planktonic nonfilamentous species and benthic and planktonic filamentous species when processing 0.1 g of Sample A with the procedure developed for the SOLID instrument (Parro *et al.*, 2011b and Experimental procedures). Results are in agreement with the habitat and cyanobacterial composition reported for the McMurdo Ice Shelf (Howard-Williams *et al.*, 1990; Fernández-Valiente *et al.*, 2001). The capacity of CYANOCHIP for efficient *in situ* detection and the broad habitat coverage from plankton, benthos, and endoliths indicate its usefulness for detecting cyanobacterial markers in planetary exploration.

### 4.3. Implications for the search for evidence of life on Mars

Our results show that even when using a best-case sample compared with what will likely be found on Mars, each analytical technique has constraints that limit the set of information produced. Further improvements in technique and instrumentation, as well as cross comparisons in varying approaches, will be required with regard to optimizing data interpretation for upcoming missions to Mars. The high fluorescence background obtained with the Raman system in the presence of phyllosilicates suggests further optimization of the technique for these types of samples. Optimizing the laser power and the signal capture with time gating will allow significant improvements. There are several factors that may impair Raman system performance even when analyzing a high organic content sample, such as the high background fluorescence caused by certain mineral phases, or the absence of the best organic targets such as the conjugated double bonds of pigments. This was the case for Sample A, where the high fluorescence background together with UV degradation of biological pigments resulted in Raman analysis barely detecting the beta-carotene signature.

Our results represent a cautionary example of the limitations of Raman spectroscopy in the detection of organic compounds on Mars, where concentrations are expected to be orders of magnitude lower. Even if organic compounds are detected, the spectral signal might be insufficient to discriminate their exact origin. Pigments are excellent targets, but samples collected for analysis must be protected from UV. It is expected that the ExoMars rover will take samples from as much as 2 m below the surface, and the Raman spectrometer will be used to attempt to decipher the mineralogy and identify any well-preserved pigment (biological or otherwise). Before deployment of such a mission, extensive work will need to be carried out to support and assess Raman system capabilities for *in situ* planetary exploration.

The use of LD-MS and GC-MS for organic and biomarker detection on Mars (Siljeström *et al.*, 2014) might be more appropriate if the known issue of cross-reactivity with perchlorate in the soils can be mitigated (Navarro-González *et al.*, 2010). However, in both cases, the biological fingerprints in the organic fraction might be lost and/or the chemical composition of the molecules modified, which would make the peak assignment and interpretation of the result complicated or even puzzling. In the best-case scenario, simple molecules could be identified, but with very little, if any, biological information (Siljeström *et al.*, 2014), as was also observed with the thermal analyses of Sample A (Fig. 3 and Table 1).

Although it was later descoped for reasons of technical maturity, an immunoassay-based sensor—the Life Marker Chip—was initially selected as part of the Pasteur payload for ExoMars (Sims *et al.*, 2005). By using our SOLID-LDChip, we rely on immunoassay to search for the biochemistry of life (Rivas *et al.*, 2008: Parro *et al.*, 2011a), and we have explored the capability of biological polymers (proteins such as antibodies, nucleic acids, and polysaccharides) to specifically interact and bind with each other in a liquid solution/suspension when searching for equal or highly similar compounds that constitute true evidence of life. Immunoassay has the limitation that the antibodies to potential martian targets must be chosen in advance from known terrestrial chemical structures for antibody production (Earth-centric approach) and the fact that martian compounds may be altered by radiation damage. We have endeavored to mitigate the former by increasing the number of antibodies and targets through a biological *shotgun* approach (multiple polyclonal antibodies to multiple targets to increase success probability) and by selecting biochemical targets that are universal in microbial structures. We can mitigate the second challenge by producing antibodies to irradiated targets and acquiring and analyzing martian samples with low exposure to radiation such as subsurface samples (McKay *et al.*, 2011).

SOLID is based on the assumption that martian life, if it ever existed, was or is based on the same biochemical principles as terrestrial life. We consider Earth biochemistry to be universal (Pace, 2001) for those life forms that evolve under similar environmental conditions. Furthermore, the transfer of material between Earth and Mars (*e.g.*, Worth *et al.*, 2013), especially during the late phase of planetary accretion when life on Earth appears to have already occurred, suggests the possibility that life on Earth and Mars may share the same biochemistry.

The LDChip probes have been produced to detect universal and evolutionarily conserved target biomarkers and compounds produced by microbes to survive extreme environments on Earth that resemble those identified on Mars. Under similar chemical conditions and temperatures, microbes are likely to respond by producing similar molecular mechanisms with which to survive. This is what happens with known life forms on Earth, where a number of extreme environments exhibit far greater differences from one another than they do from some martian counterparts. On Earth alone, biological polymers produced by microbes, regardless of environment, do not differ enough such that they cannot be recognized by immunological tools.

We tested the LDChip with samples from the deep subsurface (4 km depth in South African mines), the extremely cold and dry Antarctic and Arctic deserts, the extremely dry and salty environment of Atacama Desert, and the extreme acidic waters of río Tinto, and we have positively identified similar and identical biological polymers (Rivas *et al.*, 2008; Parro *et al.*, 2011a, b; Blanco *et al.*, 2012, 2014; this work). The antibody-based assay can only detect what it was designed to detect, that is, universal biochemical structures. It has been argued that a second life genesis on Mars or Europa could be based on a very different biochemistry, for example, D-amino acid proteins or more l-sugars, or other types of polymers (Davies and Lineweaver, 2005; Davila and McKay, 2014). It is feasible to produce antibodies to xenobiotics and other non-Earth life molecules for incorporation into an immunosensor. With the LDChip, we can address the question as to whether terrestrial-like life has occurred on Mars.

Carr *et al.* (2013) went further with regard to the search for terrestrial-like life on Mars by suggesting the development of an instrument for *in situ* DNA extraction and sequencing. DNA sequencing would be a much more Earth-centric technique for life detection and would require starting material of very high quality. The absence of cyanobacterial DNA (Fig. 4) in our Sample A, while other biomarkers are well detected with CYANCHIP, indicates how critical the appropriate sample selection is for *in situ* DNA analysis. Even if samples are returned to Earth for exhaustive analysis, proving a biological origin from ancient Mars samples would depend on finding and returning the right samples.

## 5. Conclusion

Unambiguous life detection in planetary exploration requires a suite of techniques that can provide complementary and integrative information. Herein, we demonstrate how spectroscopic, molecular, and biochemical techniques have their own limitations even when dealing with a 20% (wt/wt) organic content sample and how they can work together to obtain information from simple molecules to complex biological markers. The spectroscopic techniques are nondestructive and allow multiple-sample analysis, which makes them a good option for sample screening and selection of those of greatest interest for further analysis. However, light spectroscopy and MS-based techniques may not have the capability to identify, or may even destroy, the biological information of detected molecules. The use of a bioaffinity-based sensor such as the LDChip can definitely identify microbial biomarkers and even inform with regard to different phylotypes or habitat-associated groups.

Each technique has its own constraints and limitations on the kind of information that can be retrieved from a sample. Aspects such as the sample diagenetic stage, sample processing, or sample presentation may affect instrument performance and should be taken into account for future missions. The complementarity among varying techniques will aid in resolution of these issues and potentially provide geochemical, structural, biochemical, and even ecological information (Table 1). Results from this study will contribute to the development of a set of tests and a data library designed to support the interpretation of data generated by future missions to Mars. Our results may suggest new designs for future payloads in the search for life in the solar system.

## Abbreviations Used

BSA: bovine serum albumin
DRIFTS: diffuse reflectance infrared Fourier transform spectroscopy
ESA: European Space Agency
GC-MS: gas chromatograph–mass spectrometer
LD-MS: laser desorption–ionization mass spectrometry
LDChip: Life Detector Chip
MOMA: Mars Organic Molecule Analyzer
MS: mass spectrometry
PCR: polymerase chain reaction
SOLID: Signs Of LIfe Detector
TG: thermogravimetry
